# Ectodysplasin A regulates epithelial barrier function through sonic hedgehog signalling pathway

**DOI:** 10.1111/jcmm.13311

**Published:** 2017-08-07

**Authors:** Sanming Li, Jing Zhou, Liying Zhang, Juan Li, Jingwen Yu, Ke Ning, Yangluowa Qu, Hui He, Yongxiong Chen, Peter S. Reinach, Chia‐Yang Liu, Zuguo Liu, Wei Li

**Affiliations:** ^1^ Eye Institute of Xiamen University Xiamen Fujian China; ^2^ Medical College of Xiamen University Xiamen Fujian China; ^3^ Fujian Provincial Key Laboratory of Ophthalmology and Visual Science Xiamen Fujian China; ^4^ Wenzhou Medical University Wenzhou Zhejiang China; ^5^ School of Optometry Bloomington Indiana University Bloomington Bloomington IN USA; ^6^ Xiamen University affiliated Xiamen Eye Center Xiamen Fujian China

**Keywords:** Ectodysplasin A, epithelial barrier dysfunction, X‐linked hypohidrotic ectodermal dysplasia, tight junction

## Abstract

Ectodysplasin A (Eda), a member of the tumour necrosis factor superfamily, plays an important role in ectodermal organ development. An *EDA* mutation underlies the most common of ectodermal dysplasias, that is X‐linked hypohidrotic ectodermal dysplasia (XLHED) in humans. Even though it lacks a developmental function, the role of Eda during the postnatal stage remains elusive. In this study, we found tight junctional proteins ZO‐1 and claudin‐1 expression is largely reduced in epidermal, corneal and lung epithelia in *Eda* mutant *Tabby* mice at different postnatal ages. These declines are associated with tail ulceration, corneal pannus formation and lung infection. Furthermore, topical application of recombinant Eda protein markedly mitigated corneal barrier dysfunction. Using cultures of a human corneal epithelial cell line and *Tabby* mouse skin tissue explants, Eda up‐regulated expression of ZO‐1 and claudin‐1 through activation of the sonic hedgehog signalling pathway. We conclude that *EDA* gene expression contributes to the maintenance of epithelial barrier function. Such insight may help efforts to identify novel strategies for improving management of XLHED disease manifestations in a clinical setting.

## Introduction

Eda is a member of the tumour necrosis factor (TNF) superfamily regulating ectodermal development in various organs. In humans, germline *EDA* mutations cause a syndrome designated as XLHED characterized by the absence or malformation of multiple ectodermal appendages resulting in hypotrichosis, hypodontia, anhidrosis or hypohidrosis 1, 2, 3, 4. Spontaneous *EDA* mutant penetrance appears in other species including dog 5, cattle 6 and mouse 7.

XLHED‐afflicted individuals in their youth are at increased risk for developing severe, acute pneumonia and respiratory infections 2, 8. Respiratory infections, wheezing and recurrent sinus infections can last throughout adulthood in most cases 9, 10. XLHED patients also experience an increased prevalence of allergic diseases 11, atopic diathesis as well as chronic dermatitis throughout their lifetime 12. It was presumed that exocrine gland insufficiencies of the respiratory tract 8, 13, 14 and skin surface 10 could lead to chronic inflammatory and recurrent infection. However, the pathogenic mechanism underlying these changes remains unclear.

Ocular surface anomalies are another major category of XLHED‐related diseases. All adult XLHED patients have corneal pathology such as pannus formation 15. *Eda* mutant *Tabby* mice also experience dramatic increases in ocular surface inflammation and corneal pannus in their adult stage 16. Previous studies favour the hypothesis that keratopathy in XLHED patients results from an altered tear film lipid layer caused by a meibomian gland developmental defect 17. However, this possibility is not supported by the finding that transfection of the *Eda‐A1* gene to *Tabby* mice prevented corneal defects without restoring meibomian gland formation. This result instead indicates that *Eda* is required for maintenance of ocular surface integrity rather than meibomian gland morphogenesis 16.

The clinical manifestations of XLHED indicate that not all of the symptoms result from *EDA* control of mechanisms underlying morphogenesis. Instead this realization prompted suggestions that *EDA* may play a role in maintaining the homeostasis of organs such as skin, cornea and pulmonary tissues after the developmental stage. Clarifying the functional roles of *EDA* in maintaining normal tissue function still warrants further study.

We show here that epithelial tight junctional integrity is compromised in the cornea, skin and lung of *Tabby* mice, which was accompanied by abnormal ZO‐1 and claudin‐1 protein expression leading to epithelial barrier dysfunction. This defect markedly increases tissue susceptibility to surface pathogenic infiltration, which leads to chronic inflammation in these mice. Moreover, we found that the Eda protein regulates epithelial tight junction formation through activation of the sonic hedgehog (Shh) signalling pathway. Our findings provide new insights into pathophysiological mechanism of XLHED disease and identify a new Eda protein function unrelated to morphogenesis during the postnatal stage.

## Materials and methods

### Animals

The *EDA* mutant *Tabby* mice (C57BL/6J‐A^W‐J^‐Eda^Ta−6J^/J) and wild‐type C57BL/6J mice were purchased from the Jackson Laboratory (Bar Harbor, ME, USA). The mice were housed at the Experimental Animal Center of Xiamen University, and some were kept in a specific pathogen‐free (SPF) barrier facility, while others were kept in a conventional facility (CF) with free access to water and food. All animal protocols were performed in accordance with the Association for Research in Vision and Ophthalmology (ARVO) statement for the use of Animals in Ophthalmic and Vision Research and approved by the Animal Ethical Committee of Xiamen University. All experiments were performed in accordance with the tenets set forth in the Declaration of Helsinki.

### Haematoxylin & eosin staining

After the animals were sacrificed, the eye, skin and lung tissues were dissected and fixed in 4% paraformaldehyde overnight and embedded in paraffin. The tissue sections (5 μm) were deparaffinized and stained with haematoxylin for 1 min. and eosin for 2 min. After that, the sections were mounted using mounting medium and examined under a light microscope (Eclipse 50i, Nikon, Japan).

### Immunostaining

Cryostat sections (6 μm) of mice eyes or skin tissues or cultured cells were fixed in 4% paraformaldehyde for 20 min. The samples were washed 3 times with PBS, followed by incubation in 0.2% Triton X‐100 for 10 min. After rinsing three times with PBS for 5 min. each and pre‐incubation with 2% bovine serum albumin (BSA) for 1 hr at room temperature, sections were incubated with anti‐CD45 (sc‐52491,1:50,Santa Cruz, Dallas, TX, USA), ZO‐1 (61‐7300, 1:100, Invitrogen, Carlsbad, CA, USA), Claudin‐1 (519,000, 1:100, Invitrogen), Gli‐1 (sc‐20687, 1:50,Santa Cruz) primary antibodies at 4°C overnight. After three washes with PBS for 10 min. each, they were incubated with AlexaFluor 488‐conjugated secondary antibody (donkey anti‐rabbit, goat, rat or mouse IgG, 1:300, Life Technologies, Carlsbad, CA, USA) for 1 hr at room temperature. After three additional PBS washes for 5 min., the samples were counterstained with DAPI and then mounted for analysis under the confocal laser scanning microscope (Fluoview 1000, Olympus, Tokyo, Japan).

### Western blotting

The skin, corneal epithelium, lung tissue or cultured cells were extracted with cold lysis buffer comprising 50 mM Tris–HCl (pH 7.5), 150 mM NaCl, 1% Nonidet P‐40, 0.5% sodium deoxycholate, 0.1% SDS, and protease and phosphatase inhibitor cocktails. Equal amounts of protein extracts were subjected to electrophoresis on 8% or 10% SDS‐PAGE and then electrophoretically transferred to PVDF membrane. After blocking in 1% BSA for 1 hr, the membranes were incubated with primary antibodies ZO‐1 (61‐7300, 1:1000, Invitrogen), claudin‐1 (519,000, 1:1000, Invitrogen), Gli‐1 (sc‐20687, 1:500,Santa Cruz) and β‐actin (A3854, 1:10,000, Sigma‐Aldrich) overnight at 4°C. After three washes with Tris‐buffered saline with 0.05% Tween‐20 for 10 min. each, the membranes were incubated with HRP‐conjugated goat or rabbit anti‐mouse or rabbit IgG (1:10,000) for 1 hr. The results were visualized by enhanced chemiluminescence reagents and recorded with an imaging system (ChemiDoc XRS, Bio‐Rad, Hercules, CA, USA).

### RNA isolation and quantitative real‐time RT‐PCR analysis

Total RNA was isolated from the samples using Trizol Reagent (Invitrogen) and extracted following the manufacturer's protocol. RNA was reverse transcribed to cDNA by the ExScript RT Reagent kit (Takara Bio, Shiga, Japan). Quantitative real‐time RT‐PCR (qRT‐PCR) was performed with a StepOne Real‐Time detection system (Applied Biosystems, Foster City, CA, USA) using an SYBR Premix Ex Taq Kit (Takara Bio, Shiga, Japan). The amplification program included an initial denaturation step at 95°C for 10 min., followed by 40 cycles of 95°C for 10 sec., 57°C for 30 sec., and 75°C for 10 sec.; then, the melting curve analysis was conducted at once from 65 to 95°C. SYBR Green fluorescence was measured after each extension step, and the specificity of amplification was evaluated by melting curve analysis. The primers were designed using Primer 3 system, and they are listed in Table S1. The results of the relative quantitative real‐time PCR were analysed by the comparative threshold cycle (C_T_) method and normalized to β*‐actin* as the reference gene.

### Evaluation of sodium fluorescein staining

One microlitre of 0.1% liquid sodium fluorescein was dropped into the conjunctival sac of mice. Ninety seconds later, corneal epithelial damage was photographed and graded with a cobalt blue filter under the slit‐lamp microscope. The cornea was divided into four quadrants, which were scored, respectively, as previously described 18. Briefly, as follows: absent, 0; slightly punctate staining less than 30 spots, 1; punctate staining more than 30 spots, but not diffuse, 2; severe diffuse staining but no positive plaque, 3; positive fluorescein plaque, 4. The scores of each quadrant were added to arrive at a final grade.

### Measurement of epithelial permeability to carboxy fluorescein

Corneal epithelial barrier function was evaluated based on measurements of time dependent carboxy fluorescein penetrance. Briefly, 1 μl of 0.3% carboxy fluorescein was administered to the ocular surface; 10 min. later, the eyes were washed with 1 ml PBS to remove the residual carboxy fluorescein. After that, the animals were sacrificed with an overdose of chloral hydrate anaesthesia. The corneas were excised, and each cornea was placed in a 1.5‐ml tube containing 500 μl of PBS. The tubes were wrapped in aluminium foil to protect the solution from light and placed on an orbital shaker for 90 min. After 1 min. centrifugation, the supernatants were measured with a Gilford Fluoro IV fluorometer (Corning, Oberlin, OH, USA) to detect the concentration of carboxy fluorescein.

### Bacterial infection examination

Tabby mice and the wild‐type mice were sacrificed, and the eyeballs and lung tissues were harvested and washed three times with sterile PBS. After that, the corneas and lung tissues were dissected and homogenized with 500 μl PBS. After low‐speed centrifugation, the supernatant was coated on LB medium (1% tryptone, 0.5% yeast extract, 1% NaCl, 1.5% agar, pH7.0) plates in triplicate and incubated at 37°C overnight, the number of bacterial colonies was then counted.

### Skin tissue explants culture

The postnatal five‐day‐old mice were sacrificed and soaked in 1% iodine for 5 min., followed by three washes with PBS for 15 min. After that, the skin tissue was separated under the dissecting microscope and cut into small pieces about 5 × 5 mm size. The skin tissue explants were then placed and cultured in six‐well plates containing Defined Keratinocyte‐SFM basal medium (10744‐019, GIBCO, Carlsbad, CA, USA). Mouse recombinant Eda protein (191‐ED, R&D, Minneapolis, MN, USA), Shh protein(S0191, Sigma‐Aldrich) and Shh inhibitor cyclopamine (C4116, Sigma‐Aldrich) at different concentrations were added in different cultures. Cultures were incubated at 37°C under 5% CO_2_ and 95% humidity for 24 hrs.

### Human corneal epithelial cell culture

Human corneal epithelial (HCE) cell line was obtained from RIKEN Biosource Center (Tokyo, Japan). The basic medium for the HCE cells is DMEM/F12, containing 6% FBS, 25 mM insulin, 10 ng/ml epidermal growth factor (EGF). The conditioned medium, which contained 20 ng/ml human recombinant Eda (3944‐ED, R&D, Minneapolis, MN) or 10 μM cyclopamine was replaced when HCE cells were grown to 70% confluence. After 6‐hrs or 24‐hrs culture, the culture medium was removed and washed immediately with PBS. The RIPA buffer or Trizol buffer was added to harvest the protein or mRNA.

### pcDNA‐EDA plasmid transfection

The *EDA* cDNA was cloned into SV40‐driven pcDNA3.1 vector. The resulting plasmid was transfected into HCE cells by Lipofectamine^®^ 2000 (11668‐019, Invitrogen, Carlsbad, CA, USA) when the cells reached 70% confluence in six‐well plates. Simultaneously, the empty pcDNA3.1 vector was transfected as the negative control.

### Statistical analysis

Statistical analysis was performed with SPSS 16.0.0 (SPSS, Chicago, IL, USA). All summary data are reported as mean ± S.E.M. Comparisons between groups were performed by an unpaired, two‐tailed Student's *t*‐test. *P* ≤ 0.05 was considered statistically significant.

## Results

### Reduction of epithelial tight junctional protein expression in *Tabby* mice

**Figure 1:**
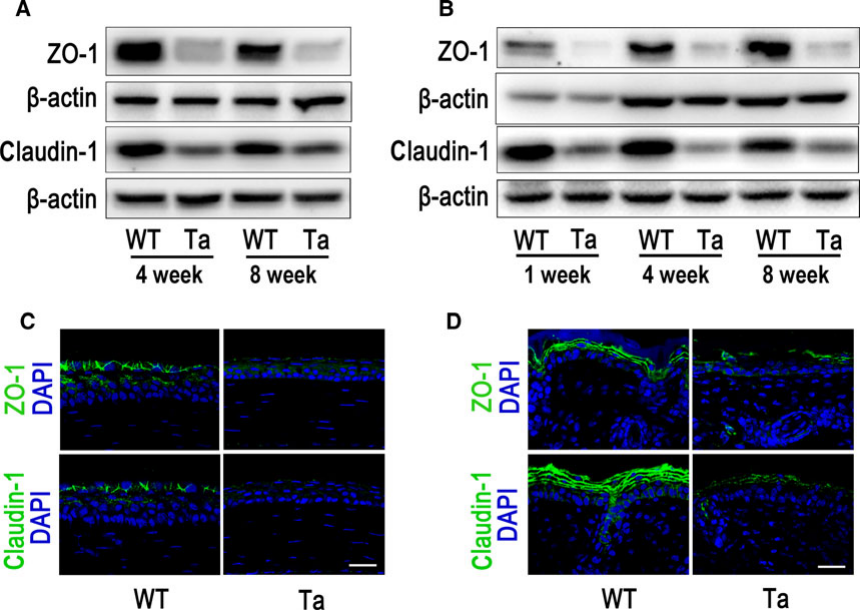
Expression of tight junction proteins, ZO‐1 and claudin‐1, in the corneal and skin epithelia of *Tabby* mice (Ta) and wild‐type mice (WT). (**A**) Western blot results show that ZO‐1 and claudin‐1 decreased in the corneal epithelia of four‐ and eight‐week‐old *Tabby* mice when compared with wild‐type control. (**B**) ZO‐1 and claudin‐1 expression was lower in the skin tissue of one‐, four‐ and eight‐week‐old *Tabby* mice. (**C**) ZO‐1 and claudin‐1 was strongly expressed in the surface layer corneal epithelium of eight‐week‐old wild‐type mice, while they were weakly expressed in *Tabby* mice. (**D**) ZO‐1 and claudin‐1 was expressed in the superficial layers of the skin epithelium of eight‐week‐old wild‐type mice, while they were weakly and discontinuously expressed in *Tabby* mice. Scale bars: 30 μm.

As the morphological changes in *Eda*‐deficient *Tabby* mice mirror those occurring in humans, this strain is frequently used to study the underlying pathophysiological mechanisms of the XLHED syndrome 19, 20. In mice, eyelid opening occurs around 2 weeks after birth. We harvested corneal epithelia, dorsal skin epithelia as well as their lung tissue from four‐ and eight‐week‐old *Tabby* mice and wild‐type littermates. Western blot results show that the corneal epithelial tight junctional ZO‐1 and claudin‐1 protein expression both dramatically decreased (Fig. 1A). Similarly such declines also occurred in their skin epithelium (Fig. 1B) and lung tissue (Fig. S1) at all time points when compared to wild‐type littermates. Immunofluorescent staining also showed discontinuous and weaker expression patterns of ZO‐1 and claudin‐1 in the corneal (Fig. 1C) and skin (Fig. 1D) epithelia of *Tabby* mice. These results suggest that there is epithelial barrier dysfunction in both neonatal and adult *Tabby* mice.

### Inflammation in corneas, skin and lung tissues of *Tabby* mice

**Figure 2:**
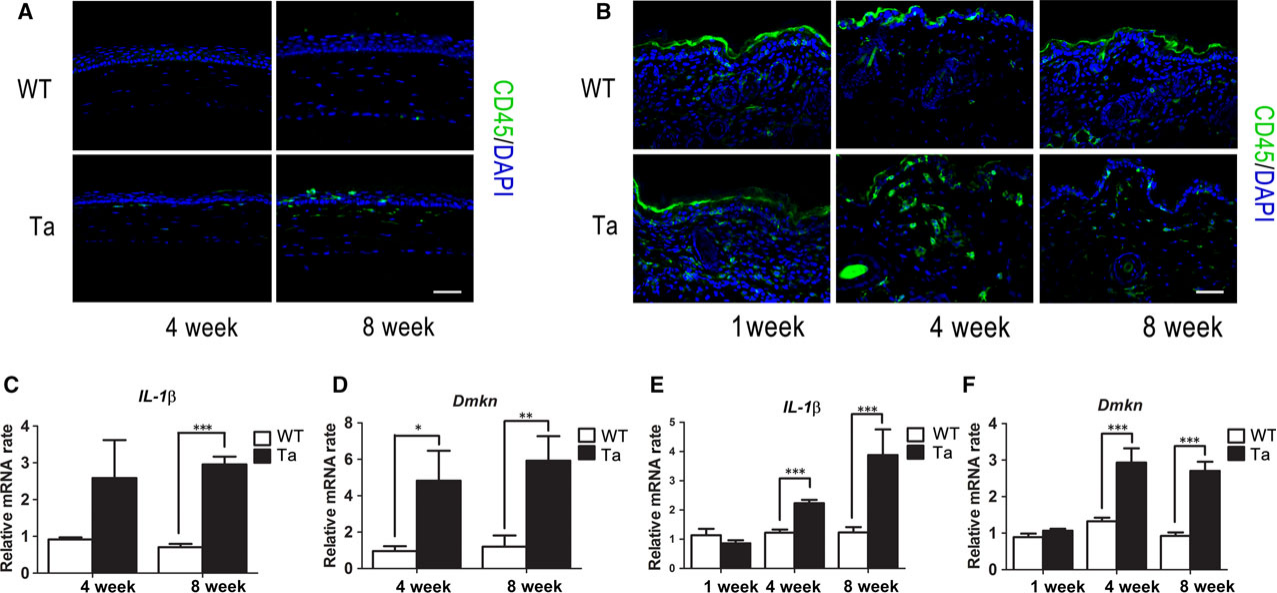
The Corneal and skin epithelial inflammation augmentation in *Tabby* mice. (**A**) Immunofluorescent staining of CD45 was negative in the wild‐type mice, while there were sporadic positive cells in the superficial corneal stroma at 4 weeks, which spread into the epithelial layer in eight‐week‐old *Tabby* mice. (**B**) CD45‐positive cells were dramatically increased in the dermis of *Tabby* mice at 4 and 8 weeks of age. The mRNA levels of *IL‐1*β (**C**) and *Dmkn* (**D**) significantly increased in four‐ and eight‐week‐old *Tabby* mice corneal epithelia when compared to the wild‐type littermates(*n* = 6 mice for each group). In skin epithelia, *IL‐1*β (**E**) and *Dmkn* (**F**) expression was also up‐regulated in four‐ and eight‐week‐old *Tabby* mice (*n* = 5 mice for each group). Data represent the mean ± S.E.M. Significance was analysed using an unpaired, two‐tailed Student's *t*‐test (*P < 0.05, **P < 0.01, ***P < 0.001). Scale bars: 60 μm.

In *Tabby* mice, ocular surface diseases occur postnatally as early as 4 weeks followed by age dependent increases in pathological severity 16. Slit‐lamp microscopy observation revealed a normal ocular surface in *Tabby* mice at 4 weeks. However, the corneal surface was scabrous and mildly oedematous in the central or peri‐central corneal stroma in eight‐week‐old *Tabby* mice (Fig. S2A). H&E staining showed that the corneal epithelial surface was irregular in *Tabby* mice at 4 weeks and 8 weeks. In contrast, the epithelial surface was smooth in wild‐type mice (Fig. S2B). Sporadic CD45‐positive macrophages were present in the anterior part of the central corneal stroma of *Tabby* mice at 4 weeks. Their numbers increased and spread into the corneal epithelium at 8 weeks, while they were absent in both layers of wild‐type littermates (Fig. 2A).

The *Tabby* mice exhibited pink skin 1 week after birth, which was maintained throughout their life (Fig. S2C). H&E staining showed mildly increased cellularity of their inter‐follicular connective tissue compared with wild‐type littermates (Fig. S2D). CD45‐positive cells dramatically increased in the skin of four‐ and eight‐week‐old *Tabby* mice (Fig. 2B), indicating chronic inflammation of the skin. We also detected in them *IL‐1*β and *Dmkn* chemokine gene expression. qRT‐PCR results showed that both chemokines very markedly increased in the corneal (Fig. 2C and D) and skin (Fig 2E and F) epithelial layers of four‐week‐old *Tabby* mice with larger rises at 8 weeks.

There was sporadic hyperaemia in one‐week‐old *Tabby* mice lung tissue along with dramatic increases in cell infiltration within the pulmonary alveoli. Immunostaining revealed numerous CD45‐positive macrophages in the lung tissue of *Tabby* mice, while they were not present in the wild‐type littermates (Fig. S3), indicating pulmonary inflammation in the neonatal *Tabby* mice.

### Corneal and pulmonary bacterial infection accompany epithelial barrier dysfunction in *Tabby* mice

**Figure 3:**
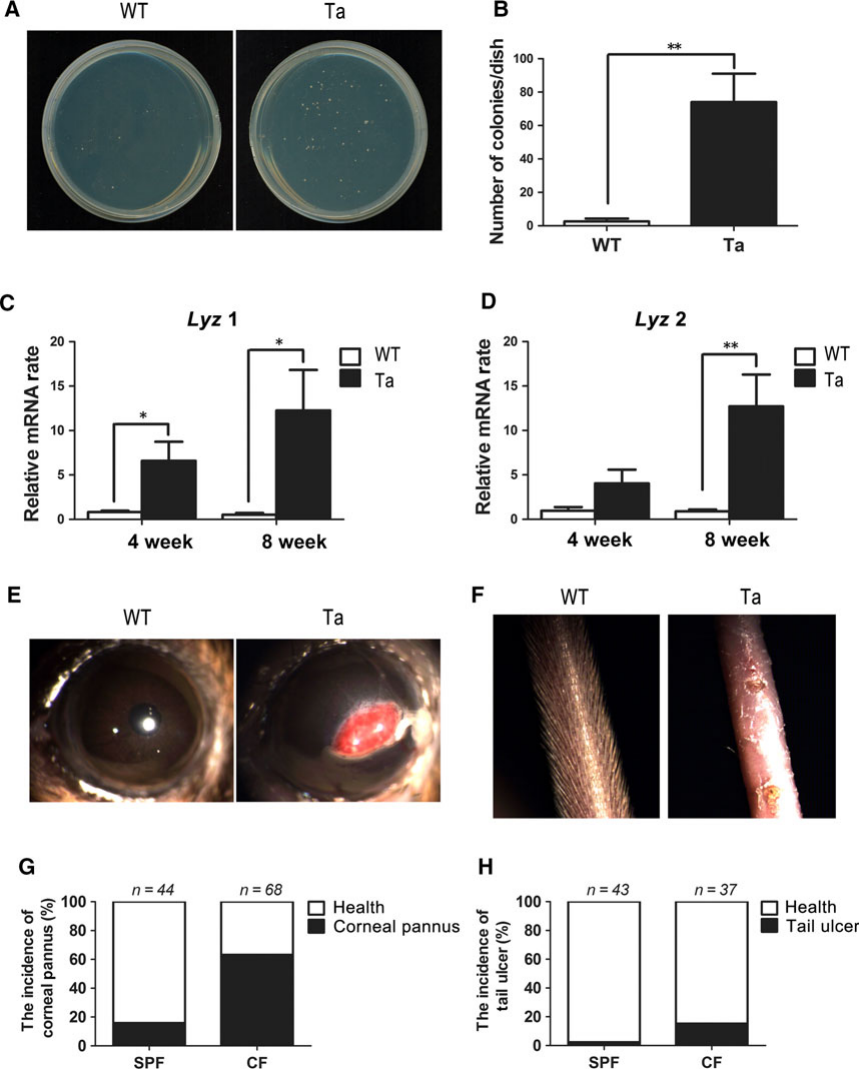
Corneal bacterial infection, corneal pannus and tail ulcer in *Tabby* mice. (**A**) Bacterial culture of the corneal tissue suspension from eight‐week‐old *Tabby* mice and wild‐type littermates. (**B**) The number of bacterial colonies was significantly larger in *Tabby* mice compared with that in wild‐type mice (*n* = 5 mice for each group). *Lysozyme 1* (*Lyz 1*) (**C**) and *Lysozyme 2* (*Lyz 2*) (**D**) mRNA expression was both up‐regulated in the corneal epithelia of four‐ and eight‐week‐old *Tabby* mice (*n* = 6 mice for each group). (**E**) Corneal pannus presented in the central cornea of 16‐week‐old *Tabby* mouse, while it was not found in wild‐type mice. (**F**) The hair of tail skin was absent in the 16‐week‐old *Tabby* mouse, and there were small ulcers present in the tail skin surface. No tail ulcer was found in wild‐type mice. (**G**) The morbidity of the corneal pannus was 15.9% in those housed in the SPF environment, while it was 63.2% in *Tabby* mice housed in the CF environment. (**H**) The morbidity of tail ulcer in *Tabby* mice was 2.4% in SPF, while it was 13.5% in CF. Data represent the mean ± S.E.M. Significance was analysed using an unpaired, two‐tailed Student's *t*‐test (*P < 0.05, **P < 0.01).

Intact corneal tight junctions are crucial for maintaining barrier function against pathogenic infiltration and preventing dysregulated immune response activation. As corneal epithelial tight junctional ZO‐1 and claudin‐1 protein expression declined in *Tabby* mice, we hypothesized that compromise of its protective barrier function would increase the possibility of bacterial infection. Accordingly, we then harvested corneal tissues from eight‐week‐old *Tabby* mice, homogenized and suspended them in PBS. Bacterial culture of the suspension from *Tabby* mice resulted in more bacterial colonies than on plates containing cultures obtained from wild‐type mice (Fig. 3A). This difference was statistically significant (Fig. 3B). Furthermore, corneal epithelial gene expression of lysozyme 1 (Fig. 3C) and lysozyme 2 (Fig. 3D) both dramatically increased in four‐ and eight‐week‐old *Tabby* mice compared with the wild‐type littermates, indicating more bacterial infection in *Tabby* mice. Similarly, bacterial culture of lung tissue suspensions from one‐week‐old *Tabby* mice was also positive while there was no bacterial colony formation from wild‐type littermate mice (Fig. S4). These differences indicate that the corneal and pulmonary inflammation was at least partly because of bacterial infection in *Tabby* mice.

To further investigate the susceptibility of *Tabby* mice to bacterial infection, we bred different groups of 16‐week‐old *Tabby* mice in either CF or SPF environments and compared the incidence of corneal pannus (Fig. 3E) and tail ulcer formation (Fig. 3F) maintained in these two different environments. The results show that the morbidity of the corneal pannus was about 63.2% for those housed in the CF while it was only 15.9% in the SPF environment (Fig. 3G). Similarly, the morbidity of tail ulcer was 13.5% in the CF environment, while it was only 2.4% in SPF environment (Fig. 3H). These differences in morbidity strongly suggest that epithelial barrier dysfunction is associated with increases in corneal bacterial infection and skin ulcer formation in *Tabby* mice.

### Recombinant Eda protein rescues corneal epithelial barrier dysfunction in *Tabby* mice

**Figure 4:**
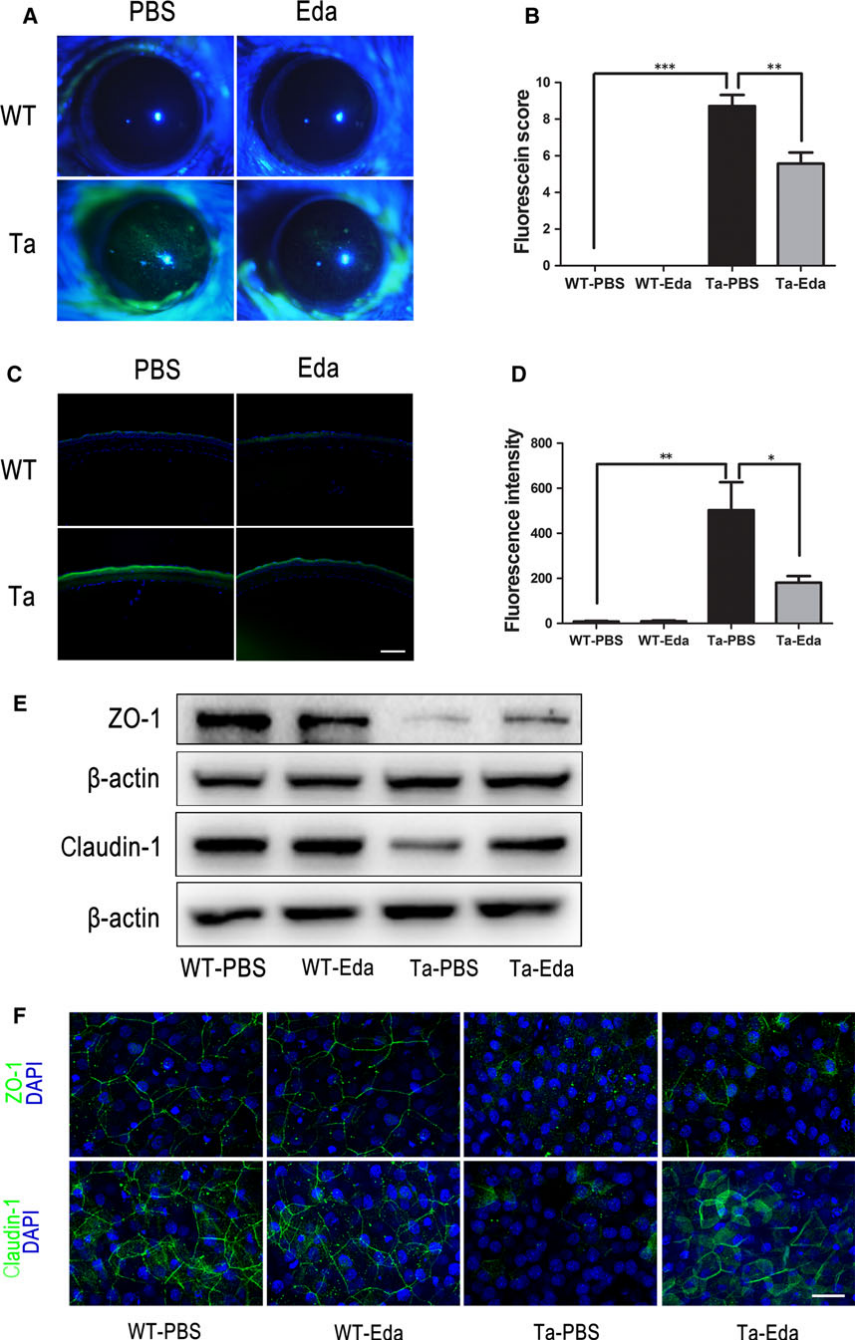
Topical Eda application partially rescues corneal epithelial barrier dysfunction in *Tabby* mice. (**A**) Wild‐type mice lack fluorescein dye staining in the cornea. *Tabby* mice cornea strongly stain in the PBS treatment group, whereas it is markedly less prominent in the Eda treatment group. (**B**) Fluorescein score dramatically decreased after Eda treatment. (**C**) Carboxy fluorescein (CF) permeation was very evident across the corneal epithelium of *Tabby* mice, while it was reduced after Eda treatment. (**D**) Fluorescence intensity significantly decreased in *Tabby* mice after Eda treatment. (**E**) Corneal epithelial ZO‐1 and claudin‐1 expression was up‐regulated after application of Eda protein in *Tabby* mice. (**F**) Whole mount staining of ZO‐1 and claudin‐1 showed both proteins were up‐regulated in the corneal epithelia of *Tabby* mice after Eda treatment. Data represent the mean ± S.E.M. Significance was analysed using an unpaired, two‐tailed Student's *t*‐test (*P < 0.05, **P < 0.01, ***P < 0.001), *n* = 4 for each group. Scale bars: 30 μm.

The Eda protein is essential for meibomian gland development 20. Interestingly, we found that the Eda protein is highly expressed in the meibomian gland and is present in human tear samples. However, its cognate receptor (Edar) is highly expressed in the corneal epithelium, but at a lower density in the meibomian gland (unpublished observation). As Eda is a secretory protein, we proposed that it could be secreted from the meibomian gland and targeted for controlling ocular surface epithelial function. One possibility is that corneal epithelial tight junctional compromise may result from an Eda deficiency because of the absence of meibomian glands in *Tabby* mice. To test whether exogenous Eda application could rescue corneal barrier dysfunction, 5 μl of a 20 ng/ml mouse recombinant Eda protein solution was administered 3 times per day to the ocular surface of four‐week‐old wild‐type and *Tabby* mice. After 3 weeks, the corneal surface fluorescein dye staining pattern clearly decreased more in *Tabby* mice relative to what occurred in *Tabby* mice treated instead with the PBS vehicle (Fig. 4A), which was confirmed by fluorescein score determination (Fig. 4B). Additional validation of this difference was obtained based on measurements in corneal epithelial carboxy fluorescein (CF) permeation. There was greater infiltration of CF in the *Tabby* mice, which declined after Eda treatment (Fig. 4C and D). On the other hand, tight junctional ZO‐1 and claudin‐1 protein expression was significantly up‐regulated by Eda protein application in the *Tabby* mice (Fig. 4E). Whole mount staining of corneal epithelia also documented ZO‐1 and claudin‐1 expression rescue in Eda treated *Tabby* mice (Fig. 4F). These results support the notion that corneal epithelial barrier dysfunction stems from the absence of Eda in *Tabby* mice.

### Eda regulates epithelial barrier function through the sonic hedgehog signalling pathway

**Figure 5:**
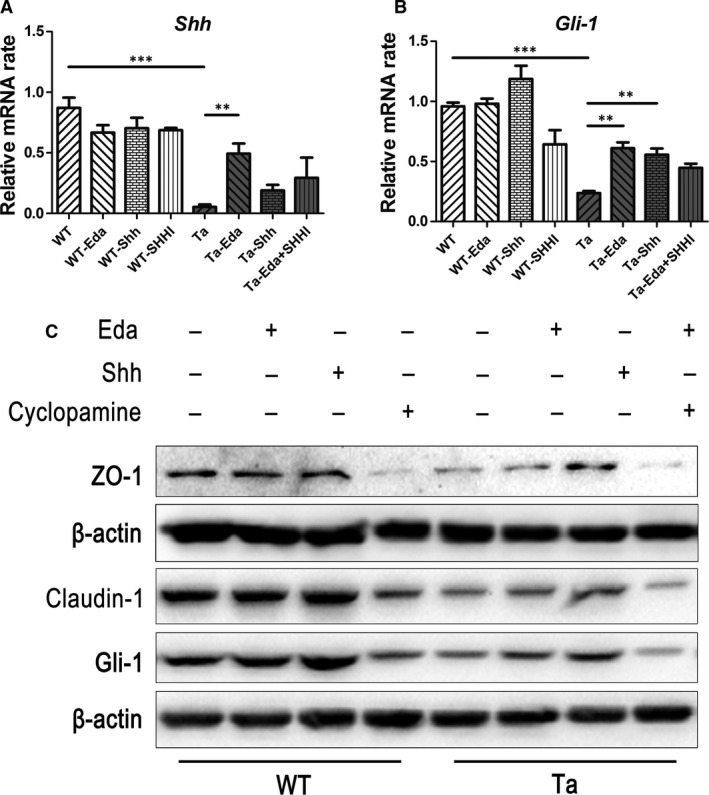
Eda activates sonic hedgehog signalling pathway in *Tabby* mice skin. One‐week‐old wild‐type and *Tabby* mice skin explants were cultured in the absence or presence of Eda (0.1 μg/ml), Shh (0.5 μg/ml), and Shh inhibitor (SHHI) cyclopamine (20 μM). Total mRNA and protein were, respectively, harvested after 24‐ and 48‐hrs culture. (**A**) The *Shh* mRNA expression in *Tabby* mice skin was much lower than that in the wild‐type mice. Addition of Eda increased the expression of *Shh*. (**B**) *Gli‐1* mRNA expression in *Tabby* mice skin was also much lower compared with wild‐type mice. Addition of Eda or Shh up‐regulated *Gli‐1* expression. (**C**) Western blot showed Shh inhibitor cyclopamine reduced expression of ZO‐1 and claudin‐1 in wild‐type skin tissue. Application of Eda or Shh protein in *Tabby* mice skin tissue culture up‐regulated ZO‐1, claudin‐1, and Gli‐1 expression. Addition of cyclopamine attenuated the effect of Eda. Data represent the mean ± S.E.M. Significance was analyzed using an unpaired, two‐tailed Student's *t*‐test (**P < 0.01, ***P < 0.001), *n* = 3 for each group.

Eda mediates its function through binding Edar and activates downstream signal cascades 21. Different studies indicate that Eda‐Edar signalling is mediated for the most part, if not totally, by the I‐κB kinase‐dependent canonical NF‐κB signalling pathway 22, 23. It was also shown that bone morphogenetic protein (BMP) activity could be suppressed while the sonic hedgehog (Shh) signalling pathway was instead up‐regulated by the Eda‐linked pathway during ectodermal organogenesis 24, 25, 26, 27, 28. Previous reports suggested that the Shh pathway regulates tight junctional protein expression 29, 30. This was validated by showing that the stomach epithelial tight junction protein expression pattern was disrupted in Shh knockout mice 31. Based on those observations, we hypothesized that Eda regulates the tight junction protein expression through the Shh signalling pathway. To test this hypothesis, skin explants from one‐week‐old wild‐type and *Tabby* mice were cultured in the absence or presence of Eda (0.1 μg/ml), Shh (0.5 μg/ml) and a Shh inhibitor cyclopamine (20 μM). After a 24‐hrs or 48‐hrs culture, total mRNA and protein harvested from the explants were analysed by qRT‐PCR and Western blot, respectively. The results show that the baseline of Shh expression was significantly lower in *Tabby* mice compared with that in wild‐type mice. Addition of Eda significantly increased the expression of Shh in *Tabby* mice skin explants, and this rise could be inhibited by cyclopamine (Fig. 5A). However, both Eda and Shh medium supplementation did not up‐regulate Shh gene expression on wild‐type mice skin explants (Fig. 5A). We also monitored *GLI‐1* gene expression, a downstream mediator in the Shh signalling pathway. *GLI‐1* was also significantly down‐regulated in *Tabby* mice. Eda and Shh application did not increase *GLI‐1* expression in wild‐type mice; however, both Eda and Shh supplementation up‐regulated *GLI‐1* mRNA expression in *Tabby* mice (Fig. 5B). Western blot results further confirmed that Eda up‐regulated Gli‐1 protein expression as well as tight junctional proteins ZO‐1 and claudin‐1, and this up‐regulation was blocked by cyclopamine (Fig. 5C).

**Figure 6:**
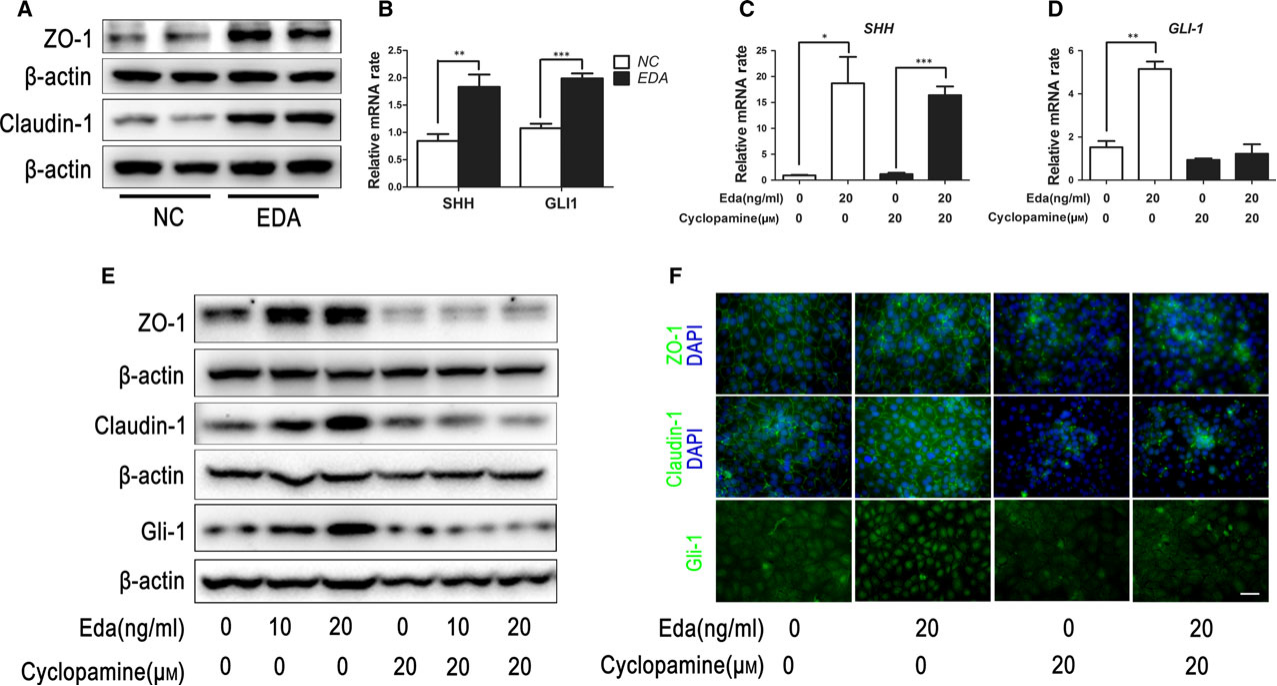
Eda regulates epithelial barrier function through the sonic hedgehog signalling pathway. (**A**) The pcDNA3.1‐EDA plasmid was transfected into human corneal epithelial (HCE) cell line for 1 week. The expression of ZO‐1 and claudin‐1 increased in EDA transfected HCE cells. (**B**) qRT‐PCR results showed *SHH* and *GLI‐1* mRNA was significantly up‐regulated in EDA transfected cells. (**C**) *SHH* gene expression in HCE cells was significantly up‐regulated by Eda protein and could not be blocked by cyclopamine. (**D**) *GLI‐1* gene expression in HCE cells was also up‐regulated by Eda protein and could be blocked by cyclopamine. (**E**) Western blot results show that Eda protein up‐regulated the expression of ZO‐1, claudin‐1 and Gli‐1 in HCE cells in a dose‐dependent manner, and this effect could be blocked by cyclopamine. (**F**) Immunofluorescent staining showed ZO‐1 and claudin‐1 expression in HCE cells was remarkably inhibited by cyclopamine. Nuclear expression of Gli‐1 was enhanced in HCE cells after Eda treatment and was down‐regulated with additional cyclopamine. Data represent the mean ± S.E.M. Significance was analysed using an unpaired, two‐tailed Student's *t*‐test (*P < 0.05, **P < 0.01, ***P < 0.001), *n* = 3 for each group. Scale bar: 60 μm.

To further validate the contribution made by Eda in regulating epithelial tight junctional protein expression and integrity, we constructed a pcDNA3.1‐EDA plasmid and transfected it into a human corneal epithelial (HCE) cell line whose *EDA* mRNA expression level was very low. One week after transfection, qRT‐PCR results showed that the *EDA* plasmid was successfully transfected as *EDA* expression dramatically increased in HCE cells (Fig. S5). As expected, ZO‐1 and claudin‐1 tight junctional protein expression also significantly increased in *EDA* transfected HCE cells (Fig. 6A). Moreover, both *SHH* and *GLI‐1* gene expression were up‐regulated in these *EDA* transfected cells (Fig. 6B).

To further document the functional role of Eda, we also applied human Eda recombinant protein to the HCE cells. Eda protein application dramatically up‐regulated *SHH* gene expression, whereas cyclopamine had no effect on this response (Fig. 6C). Similarly, *GLI‐1* gene expression was also up‐regulated by Eda, while cyclopamine markedly decreased this rise (Fig. 6D). Western blot results showed that Eda dose dependently up‐regulated ZO‐1, claudin‐1 and Gli‐1 expression, and such an effect could be blocked by cyclopamine (Fig. 6E). Immunofluorescent staining further confirmed that ZO‐1 and claudin‐1 was strongly enhanced in HCE cells after Eda treatment, while cyclopamine remarkably inhibited the expression of these proteins. Meanwhile, Gli‐1 nuclear expression was dramatically induced by Eda and could be blocked by cyclopamine (Fig. 6F).

## Discussion

*Eda* mRNA is expressed in various organs and tissues, including the heart, kidney, pancreas, brain, lung, liver, skeletal muscle, teeth, as well as the skin during both embryonic development and adulthood 1, 32. Even though it is known that the *Eda* gene has a developmental function in controlling the morphogenesis of various ectodermal structures such as hair, teeth, nails and exocrine glands, little is known about its postnatal function. In the current study, for the first time, we found tight junction proteins such as ZO‐1 and claudin‐1 were dramatically down‐regulated in *Eda* mutant *Tabby* mice, resulting in epithelial barrier dysfunction in various tissues such as skin, cornea and lung. We further proved that it is Eda that makes an essential contribution to maintaining surface epithelial homeostasis through regulation of tight junction formation.

Epithelial apical tight junctional integrity plays a critical role in providing an epithelial barrier function against pathogenic infiltration. Its intactness is also needed for cell–cell communication and establishing cell polarity as well as regulating paracellular movement of ions and macromolecules 33. Disruption of the tight junction is characteristic of a number of diseases including dermatitis, pathogenic infection and cancer 34. It was proposed long ago that genetically impaired epidermal barrier formation may be the primary cause of the rapid increase in the prevalence of atopic dermatitis and respiratory atopy 35. Recently, defective expression of epidermal ZO‐1 and claudin‐1 was observed in the skin of patients with atopic dermatitis 36, where such pathology is a risk factor for viral infection and allergen sensitization 37, 38. Our results clearly demonstrate that compromise of tight junctional integrity is associated with marked increases in vulnerability to bacterial infection and chronic inflammation in *Tabby* mice. Losses in junctional compaction may help explain why XLHED patients are at a greater of risk of suffering from pneumonia, respiratory infections and are also more frequently afflicted with allergic diseases, chronic dermatitis and ocular surface inflammation than the general population.

Although the Eda‐Edar signalling pathway was intensively studied, most studies focused on the effect of *Eda* linked signalling pathway activation on morphogenesis 3. In our study, we found that Eda regulates ZO‐1 and claudin‐1 tight junctional protein expression through activation of Shh signalling pathway using *in vivo* and *in vitro* methodology. It was reported that the stomach epithelial cell–cell junction was disrupted in *Shh* conditional knockout mice 31, which also supports the notion that *Shh* is situated downstream from *Eda* in this signalling pathway.

Recombinant Eda protein injection into pregnant *Tabby* mouse could reverse the XLHED phenotype in the offspring 39. Similarly administration of Fc‐Eda to dog models of XLHED during their first 2 weeks of life also rescued developmental abnormalities 40, 41. Animals treated with recombinant Eda protein in prenatal or early postnatal stage showed dramatically decreased, if not all, clinical signs such as eye and airway infections with improved lacrimation, meibomain gland and bronchial glands formation 40, 41. Therefore, the ocular surface defect and pulmonary infection in EDA mutant animals may synergistically resulted from both secretory glands defect and the absence of biological function of EDA. In other words, normal secretory glands in the ocular surface and pulmonary tissues may compensate the deficiency of EDA. However, this interpretation does not fit previous study which showed transfection of the Eda‐A1 gene to *Tabby* mice prevented corneal defects without restoring meibomian gland formation 16. To further address this contradiction, inducible Eda knockout mice should be generated to knock down EDA gene in the adult stage, allowing us to investigate the function of Eda during the adult stage while eliminating any involvement of morphogenesis.

Currently, phase 2 neonate clinical trials are being conducted using humanized Fc‐Eda‐A1 protein, EDI200 to treat XLHED (www.clinicaltrials.gov, NCT01775462). There is optimism that EDI200 treatment will correct the developmental disorders of XLHED in humans. We tested if recombinant Eda protein application to the ocular surface of postnatal *Tabby* mice reverses declines in epithelial barrier tightness. This procedure largely rescued ZO‐1 and claudin‐1 tight junction protein expression. Our results point to the possibility that repeated exogenous Eda treatment may be necessary to sustain this protein at high enough levels to maintain normal tight junction barrier function from childhood into adulthood in XLHED patients. In summary, *EDA* gene expression provides essential support for the maintenance of epithelial tight junctional integrity during postnatal stage. This effect is of importance in hindering pathogenic infiltration and protecting tissues from incurring declines in their normal function. As such control by *EDA* gene expression of barrier function is mediated through the Shh signalling pathway, this finding pinpoints another potential drug target for treating XLHED‐related diseases.

## Conflict of interests

The authors have declared that no competing interests exist.

## Supporting information


**Figure S1** ZO‐1 and claudin‐1 expression in lung tissue of *Tabby* mice.
**Figure S2** Inflammation of skin tissue in *Tabby* mice.
**Figure S3** Inflammation of lung tissue in *Tabby* mice.
**Figure S4** Pulmonary bacterial infection in *Tabby* mice.
**Figure S5** EDA plasmid transfection in HCE cells.
**Table S1** Primer sequence pairs used for quantitative real‐time PCR.
