# Supplementary material for: Ectodysplasin A regulates epithelial barrier function through sonic hedgehog signalling pathway
Source: J Cell Mol Med. 2017 Aug 7;22(1):230–40. doi: 10.1111/jcmm.13311 (PMC5742694; doi:10.1111/jcmm.13311)
Supplement: Supplementary file 1 — Figure S1 ZO‐1 and claudin‐1 expression in lung tissue of Tabby mice. Figure S2 Inflammation of skin tissue in Tabby mice. Figure S3 Inflammation of lung tissue in Tabby mice. Figure S4 Pulmonary bacterial infection in Tabby mice. Figure S5 EDA plasmid transfection in HCE cells. Table S1 Primer sequence pairs used for quantitative real‐time PCR. [file JCMM-22-230-s001.docx]

**Supplementary Information**


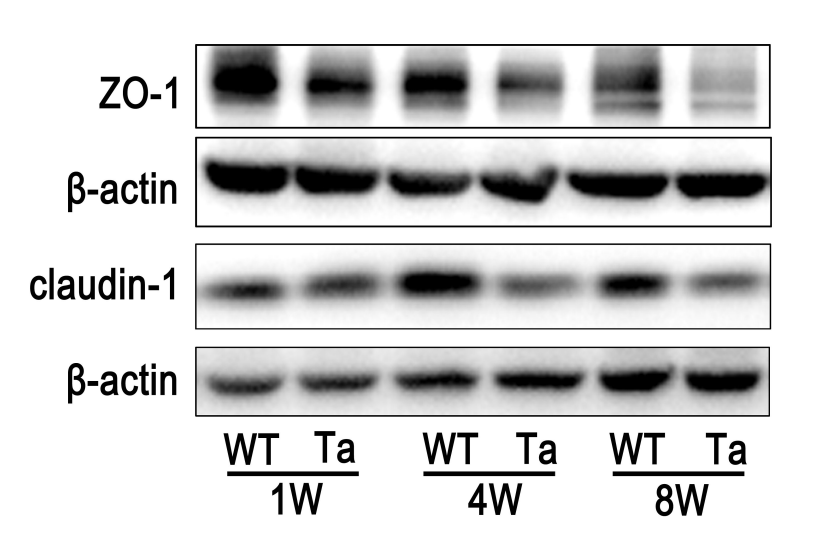


**Figure S1. ZO-1 and claudin-1 expression in lung tissue of *Tabby* mice.** Western blot results showed that ZO-1 and claudin-1 decreased in lung of 1, 4 and 8 weeks old *Tabby* mice when compared with wild-type littermates.


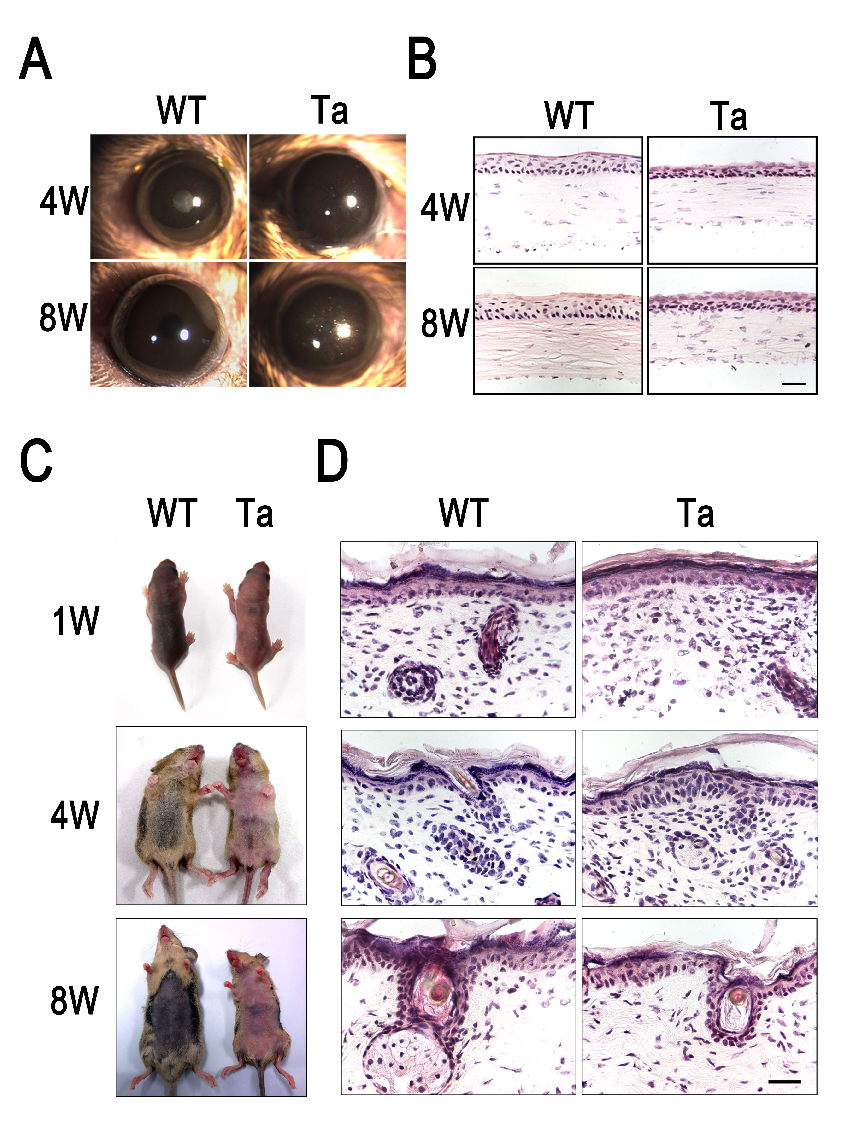


**Figure S2. Inflammation of skin tissue in *Tabby* mice.** **(A)** Corneal surface was smooth in the wild type mice at 4 and 8 weeks. However, there was scabrous corneal surface and mild edematous of corneal stroma at 8 weeks *Tabby* mice. **(B)** H&E staining of corneal tissues showed smooth epithelial surface in the wild type mice, while showed jagged surface in *Tabby* mice. **(C)** The pink skin was shown in 1, 4 and 8 weeks old *Tabby* mice when compared with wild type littermates. **(D)** H&E staining showed increased cellularity of dermis tissue in *Tabby* mice.


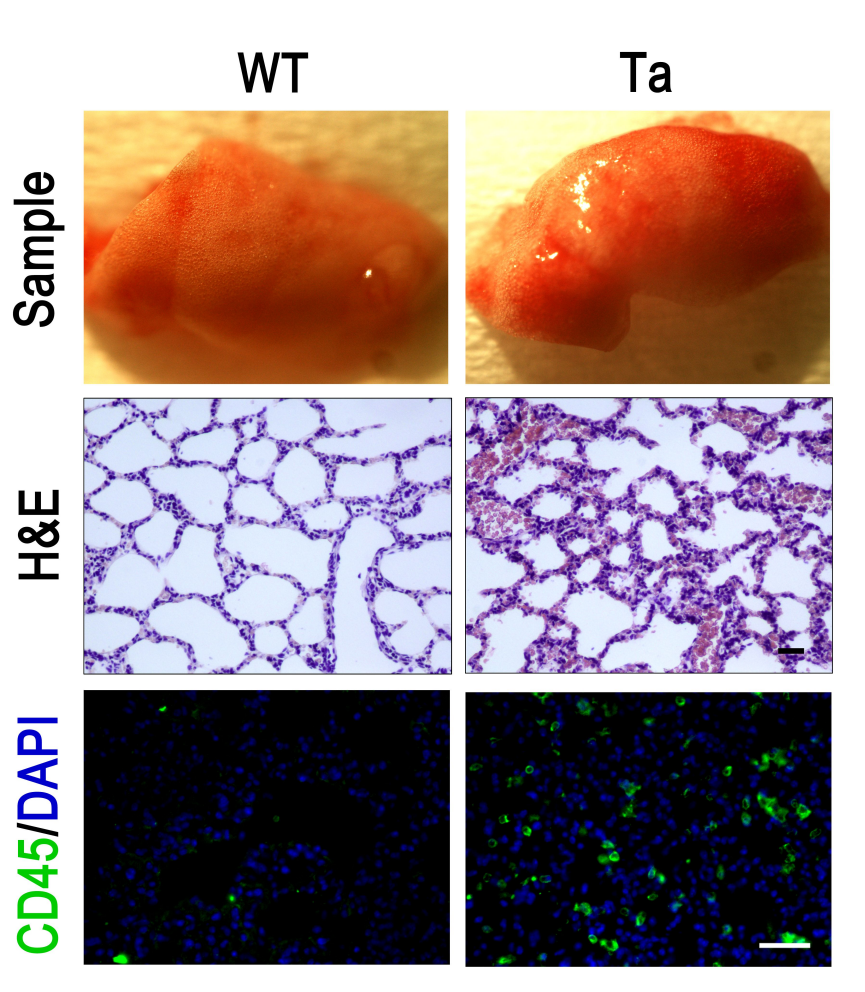


**Figure S3. Inflammation of lung tissue in *Tabby* mice.** The lung tissue from 1 week old *Tabby* mice showed sporadic hyperaemia. H&E staining showed dramatic increase of cell infiltration among and within the pulmonary alveoli. Immunostaining revealed numerous CD45 positive cells presented in the lung tissue of *Tabby* mice, while was absent in the wild-type littermates. Scale bars: 60μm.


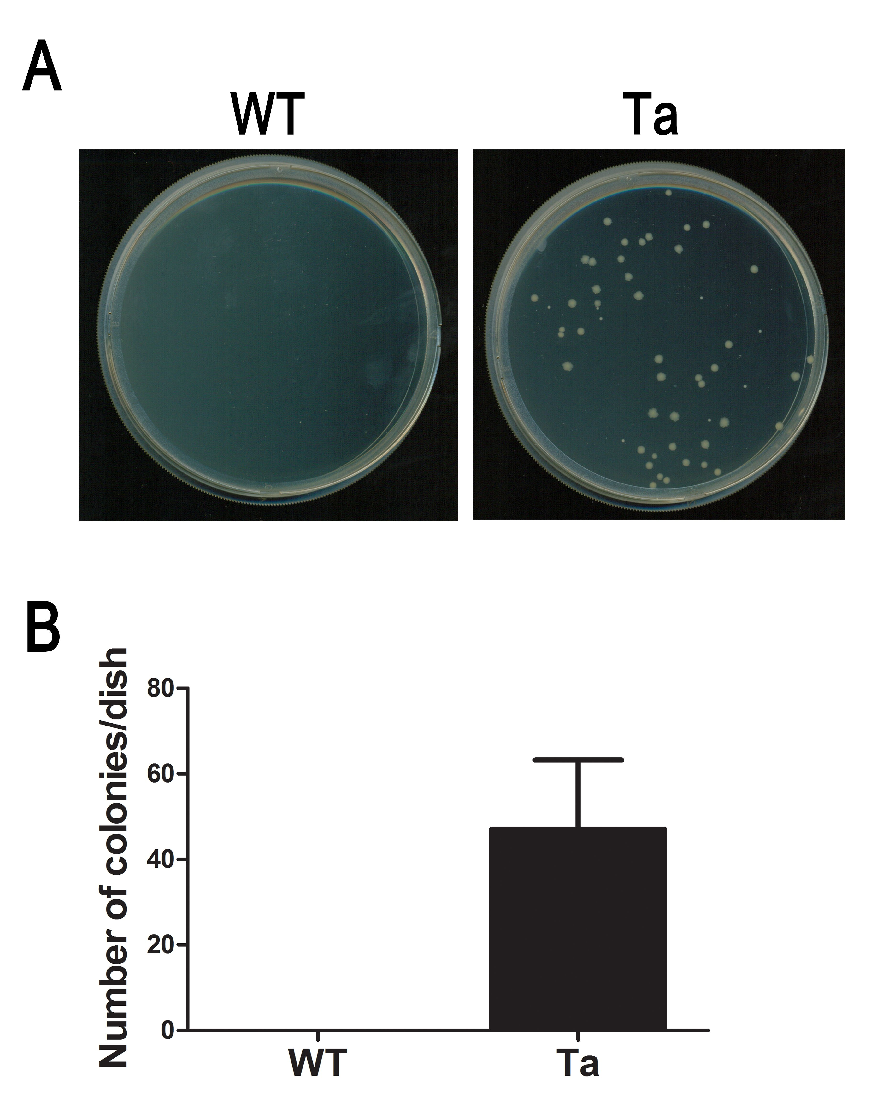


**Figure S4. Pulmonary bacterial infection in *Tabby* mice. (A)** Bacterial culture of the lung tissue suspension from 1 week old *Tabby* mice showed positive bacteria colonies, while there was no colony formation from wild type littermates. **(B)** The number of colonies was counted and compared in wild type mice and *Tabby* mice group (n=4 for each group). Data represent the mean ± SEM.


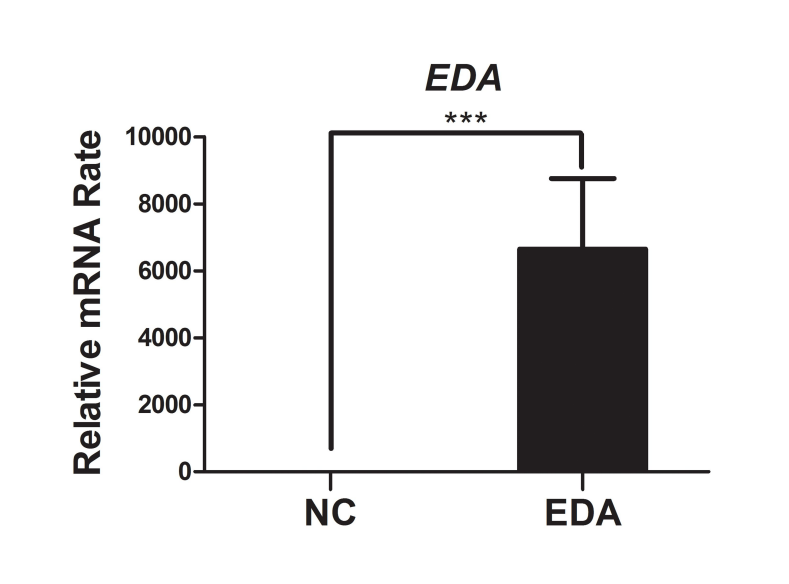


**Figure S5. EDA plasmid transfection in HCE cells.** qRT-PCR results showed that EDA mRNA was highly expressed in HCE cells after transfection of the pcDNA3.1-EDA plasmid, while the negative control showed very low level of EDA mRNA expression. Data represent the mean ± SEM. Significance was analyzed using an unpaired, 2-tailed Student’s t test (***P < 0.001), n=5 for each group.

**Table S1. Primer sequence pairs used for quantitative real-time PCR.**

| **Gene** | **Sense** | **Antisense** |
| --- | --- | --- |
| ***Actb*** | 5’-CCTAAGGCCAACCGTGAAAAG-3’ | 5’-AGGCATACAGGGACAGCACAG-3’ |
| ***IL-1β*** | 5’-GGGCCTCAAAGGAAAGAATC-3’ | 5’-TACCAGTTGGGGAACTCTGC-3’ |
| ***Dmkn*** | 5’-GAAGGCTATTCGGTCTCCAGG-3’ | 5’-GAGCCTCCAAGGAGATGACTG-3’ |
| ***Lyz-1*** | 5’-TGGAATGGATGGCTACCGTG-3’ | 5’-CCATAGTCGGTGCTTCGGTC-3’ |
| ***Lyz-2*** | 5’-GGAATGGCTGGCTACTATGG-3’ | 5’-TTGGTCTCCACGGTTGTAGT-3’ |
| ***Shh*** | 5’-GATGAGGAAAACACGGGAGCAG-3’ | 5’-CCACTGCTCGACCCTCATAGT-3’ |
| ***Gli-1*** | 5’-ACAGTGAGCATATCCACGGG-3’ | 5’-CGAAGGTGCGTCTTGAGGTT-3’ |
| ***ACTB*** | 5’-TGACGTGGACATCCGCAAAG-3’ | 5’-CTGGAAGGTGGACAGCGAGG-3’ |
| ***EDA*** | 5’-GGACGGCACCTACTTCATCT-3’ | 5’-GGAATCTAGGATGCAGGGGC-3’ |
| ***SHH*** | 5’-TGAAAGCAGAGAACTCGGTGG-3’ | 5’-CAGGAAAGTGAGGAAGTCGCT-3’ |
| ***GLI-1*** | 5’-CAGGGAGGAAAGCAGACTGA-3’ | 5’-ACTGCTGCAGGATGACTGG-3’ |
